# Commentary: Relationships between mathematics performance and attitude to mathematics

**DOI:** 10.3389/fpsyg.2023.1124571

**Published:** 2023-04-17

**Authors:** Fabienne Crettaz von Roten

**Affiliations:** Faculty of Social and Political Sciences, Institute of Sport Sciences, University of Lausanne, Vaud, Switzerland

**Keywords:** sample size, power, teaching, reviewing, psychology

## Introduction

Psychology is one of the scientific fields in which the dangers of publications based on a small number of subjects have been commented on very early and comprehensively: see for example Cohen's (1969) work on sample size calculation in the late 60s and Maxwell's (2004) report on the low power of many psychological studies due to insufficient sample size. In this context, I was surprised to read an article in *Frontiers Educational Psychology* published in March 2022 regarding the results of a 2 × 2 factorial design with 10 subjects per cell (Dowker and Sheridan, 2022).

## The problem

The authors did not provide calculations of sample size. However, for a power of 0.80, a type I error of 0.05 and a medium size effect, 128 subjects are needed for a two-way ANOVA or for a multiple linear regression with three predictors (the other analysis the authors conducted) a total of 77 subjects. Since in the context of educational psychology, a large size effect is very unlikely, this study with 40 subjects is more than “somewhat underpowered” (p. 6). The authors resolve the problem by doing frequentist and Bayesian inference, which means that they double the number of statistical tests performed. This is not really the solution because the global type I error increases drastically.

## Discussion

Please do not misunderstand the purpose of this commentary: it is not meant as a reproach to the authors but as a reflection on solutions.

It seems that the teaching of statistics and methodology in the cursus of psychology is insufficiently focused on sample size issues because this problem still arises in *Frontiers* and other psychological reviews. There should be a rethinking of how instructors teach this aspect in higher education, i.e. move more on conceptual issues and consequences than on technicalities. Focusing on real data, Spiegelhalter (2019) perfectly clarified the importance of sample size.

Then, it is the duty of a scientific journal's editors in chief to apply a minimum of statistical reviewing because they act as gatekeepers of scientific quality. First, they could edit guidelines for statistical reporting to help authors improve it. The American Psychological Association (2020) manual is very clear on the issue of sample size, but this matter is perhaps lost in the more than 700 pages of its seventh edition. Therefore, reviews need guidelines for authors – inspired by those of high-quality reviews – that involve the key statistical elements that are important for the study's validity (sample size, verification of the conditions for applying a statistical analysis, etc). These guidelines could also mention books or articles on this topic, that could help the authors on this issue. Second, a statistical review should be conducted by the editorial team or by qualified statisticians recruited as reviewers – which is done in some medical reviews – or by selecting at least one reviewer with strong statistical skills, who would receive the task of paying specific attention to statistical aspects. Finally, as Altman (2002) suggested, reviews could encourage letters adopting a critical approach to statistical reporting in published articles.

## Author contributions

The author confirms being the sole contributor of this work and has approved it for publication.
